# Supplementary material for: Improved inference of population histories by integrating genomic and epigenomic data
Source: eLife. 2024 Sep 12;12:RP89470. doi: 10.7554/eLife.89470 (PMC11392530; doi:10.7554/eLife.89470)
Supplement: Supplementary file 1. — (a) Average mean root square error (MRSE) of demographic inference in Figure 2, Figure 2—figure supplement 1 and Figure 2—figure supplement 2. Average mean root square error (in log10) of demographic inference in Figure 2A–D, Figure 2—figure supplements 1 and 2 shows the three approaches (eSMC2, SMCtheo with unknown rates, SMCtheo with known rates and MSMC2). The coefficient of variation is indicated in parentheses (b) Percentage of repetitions rejecting the H0 hypothesis at P=0.05 of binomial distribution of epimutations over 100 repetitions using two sequences of 100 Mb with recombination and mutation rate set to 1×10^−8^ per generation per bp under a constant population size fixed to 10,000. (c) Average estimated rate of the site methylation and demethylation rates from simulations. True versus average estimated values of the site methylation and demethylation rates over ten repetitions. We use two sequences of 100 Mb with r=μ1=10−8 per generation per bp under a constant population size fixed to 10,000. The coefficient of variation is indicated in brackets. (d) Average estimated rate of the region methylation and demethylation rates from simulations. True versus average estimated values of the region methylation and demethylation rates over ten repetitions. We use two sequences of 100 Mb with r=μ1=10−8 per generation per bp under a constant population size fixed to 10,000. The coefficient of variation is indicated in brackets. (e) Average estimated rate of both site and region methylation and demethylation rates from simulations. Average estimated values of the site and region methylation and demethylation rates over ten repetitions using 2 sequences of 100 Mb with recombination and mutation rate set to 1×10^−8^ per generation per bp under a constant population size fixed to 10,000. The coefficient of variation is indicated in brackets. (f) Average mean root square error of demographic inference in Figure 5. Average mean root square error (in log10) of demographic in [file elife-89470-supp1.pdf]

# Improved inference of population histories by integrating genomic and epigenomic data - Supplementary File 1

**Thibaut Sellinger[1,2], Frank Johannes[3], Aurélien Tellier[1*]**

***For correspondence:**
aurelien.tellier@tum.de (AT)

[1]Professorship for Population Genetics, Department of Life Science Systems, Technical University of Munich; [2]Department of Environment and Biodiversity, Paris Lodron University of Salzburg; [3]Professorship for Plant Epigenomics, Department of Molecular Life Sciences, Technical University of Munich

**Supplementary File 1a.** Average mean root square error (MRSE) of demographic inference in Figure 2, Figure 2 - sup. 1 and Figure 2 - sup. 2. Average mean root square error (in log10) of demographic inference in Figure 2A-D, Figure 2 - sup. 1 and 2 shows the three approaches (eSMC2, SMCtheo with unknown rates, SMCtheo with known rates and MSMC2). The coefficient of variation is indicated in parentheses

| Approach | MRSE Fig 2A | MRSE Fig 2B | MRSE Fig 2C | MRSE Fig 2D | MRSE Fig 2 Sup1 | MRSE Fig 2 Sup2 |
|---|---|---|---|---|---|---|
| eSMC2 | 6.92 | 5.41 | 8.68 (0.020) | 9.17 | 6.92 | 7.12 (0.01) |
| SMCtheo (known rates) | 6.42 | 6.00 | 6.77 (0.004) | 8.74 | 6.73 | 6.90 (0.02) |
| SMCtheo (unknown rates) | 6.60 | 5.98 | | 10.59 | 6.73 | |
| MSMC2 | 7.02 | 10.1 | 11.76 (0.04) | 10.74 | 6.83 | |

**Supplementary File 1b.** Percentage of repetitions rejecting the $H_0$ hypothesis at p=0.05 of binomial distribution of epimutations over 100 repetitions using two sequences of 100 Mb with recombination and mutation rate set to $1 \times 10^{-8}$ per generation per bp under a constant population size fixed to 10,000.

| Region methylation rate | Region demethylation rate | Site methylation rate | Site demethylation rate | % of repetitions accepting region epimutations |
|---|---|---|---|---|
| $10^{-2}$ | $5 \times 10^{-2}$ | $0$ | $0$ | 100% |
| $10^{-4}$ | $5 \times 10^{-4}$ | $0$ | $0$ | 100% |
| $10^{-6}$ | $5 \times 10^{-6}$ | $0$ | $0$ | 100% |
| $10^{-8}$ | $5 \times 10^{-8}$ | $0$ | $0$ | 100% |
| $0$ | $0$ | $1 \times 10^{-8}$ | $5 \times 10^{-8}$ | 2% |
| $0$ | $0$ | $1 \times 10^{-6}$ | $5 \times 10^{-6}$ | 1% |
| $0$ | $0$ | $1 \times 10^{-4}$ | $5 \times 10^{-4}$ | 0% |
| $0$ | $0$ | $1 \times 10^{-2}$ | $5 \times 10^{-2}$ | 0% |
| $10^{-4}$ | $5 \times 10^{-4}$ | $1 \times 10^{-8}$ | $5 \times 10^{-8}$ | 100% |
| $10^{-4}$ | $5 \times 10^{-4}$ | $1 \times 10^{-6}$ | $5 \times 10^{-6}$ | 100% |
| $10^{-4}$ | $5 \times 10^{-4}$ | $1 \times 10^{-4}$ | $5 \times 10^{-4}$ | 100% |
| $10^{-4}$ | $5 \times 10^{-4}$ | $1 \times 10^{-2}$ | $5 \times 10^{-2}$ | 0% |
| $10^{-4}$ | $5 \times 10^{-4}$ | $5 \times 10^{-8}$ | $1 \times 10^{-8}$ | 100% |
| $10^{-4}$ | $5 \times 10^{-4}$ | $5 \times 10^{-6}$ | $1 \times 10^{-6}$ | 100% |
| $10^{-4}$ | $5 \times 10^{-4}$ | $5 \times 10^{-4}$ | $1 \times 10^{-4}$ | 100% |
| $10^{-4}$ | $5 \times 10^{-4}$ | $5 \times 10^{-2}$ | $1 \times 10^{-2}$ | 0% |

**Supplementary File 1c.** Average estimated rate of the site methylation and demethylation rates from simulations. True versus average estimated values of the site methylation and demethylation rates over ten repetitions. We use two sequences of 100 Mb with $r = \mu_1 = 10^{-8}$ per generation per bp under a constant population size fixed to 10,000. The coefficient of variation is indicated in brackets.

| True site methylation rate | Estimated site methylation rate | True site demethylation rate | Estimated site demethylation rate |
|---|---|---|---|
| $10^{-8}$ | $1.0 \times 10^{-8}$ (0.03) | $5 \times 10^{-8}$ | $5.0 \times 10^{-8}$ (0.03) |
| $10^{-6}$ | $1.10^{-6}$ (0.01) | $5 \times 10^{-6}$ | $5.3 \times 10^{-6}$ (0.01) |
| $10^{-4}$ | $1.6 \times 10^{-4}$ (0.05) | $5 \times 10^{-4}$ | $8 \times 10^{-4}$ (0.05) |
| $10^{-2}$ | $3.6 \times 10^{-3}$ (0.74) | $5 \times 10^{-2}$ | $1.8 \times 10^{-2}$ (0.74) |

**Supplementary File 1d.** Average estimated rate of the region methylation and demethylation rates from simulations. True versus average estimated values of the region methylation and demethylation rates over ten repetitions. We use two sequences of 100 Mb with $r = \mu_1 = 10^{-8}$ per generation per bp under a constant population size fixed to 10,000. The coefficient of variation is indicated in brackets.

| True region methylation rate | Estimated region methylation rate | True region demethylation rate | Estimated region demethylation rate |
|---|---|---|---|
| $10^{-8}$ | $6.3 \times 10^{-9}$ (0.02) | $5 \times 10^{-8}$ | $3^{-8}$ (0.02) |
| $10^{-6}$ | $1.1^{-6}$ (0.03) | $5 \times 10^{-6}$ | $5.4 \times 10^{-6}$ (0.03) |
| $10^{-4}$ | $1.6 \times 10^{-4}$ (0.16) | $5 \times 10^{-4}$ | $7.8 \times 10^{-4}$ (0.15) |
| $10^{-2}$ | $2.6 \times 10^{-3}$ (0.77) | $5 \times 10^{-2}$ | $1.4 \times 10^{-2}$ (0.77) |

**Supplementary File 1e.** Average estimated rate of both site and region methylation and demethylation rates from simulations. Average estimated values of the site and region methylation and demethylation rates over ten repetitions using 2 sequences of 100 Mb with recombination and mutation rate set to $1 \times 10^{-8}$ per generation per bp under a constant population size fixed to 10,000. The coefficient of variation is indicated in brackets.

| Methylation rate | Estimated methylation rate | Demethylation rate | Estimated demethylation rate |
|---|---|---|---|
| $10^{-8}$ | $8.0 \times 10^{-9}$ (0.04) | $5 \times 10^{-8}$ | $5.2 \times 10^{-8}$ (0.04) |
| $10^{-6}$ | $1.3 \times 10^{-6}$ (0.01) | $5 \times 10^{-6}$ | $8.0 \times 10^{-6}$ (0.01) |
| $10^{-4}$ | $9.5 \times 10^{-4}$ (0.19) | $5 \times 10^{-4}$ | $5.1 \times 10^{-3}$ (0.19) |
| $10^{-2}$ | $8.5 \times 10^{-2}$ (0.85) | $5 \times 10^{-2}$ | $4.6 \times 10^{-1}$ (0.85) |
| Region methylation rate | Estimated region methylation rate | Region demethylation rate | Estimated region demethylation rate |
| $10^{-8}$ | $2.9 \times 10^{-9}$ (0.37) | $5 \times 10^{-8}$ | $2.6 \times 10^{-8}$ (0.37) |
| $10^{-6}$ | $8.4 \times 10^{-7}$ (0.03) | $5 \times 10^{-6}$ | $8.4 \times 10^{-6}$ (0.03) |
| $10^{-4}$ | $2.5 \times 10^{-4}$ (0.38) | $5 \times 10^{-4}$ | $3.0 \times 10^{-3}$ (0.38) |
| $10^{-2}$ | $2.3 \times 10^{-3}$ (0.22) | $5 \times 10^{-2}$ | $2.9 \times 10^{-2}$ (0.22) |

**Supplementary File 1f.** Average mean root square error of demographic inference in Figure 5. Average mean root square error (in log10) of demographic inference in Figure 5 by the two approaches eSMC2, SMCm with unknown epimutations rates (A and C), and SMCm with known epimutation rates (B and D). Note the second row indicates the MRSE in recent times (younger than 400 generations ago). The coefficient of variation is indicated in parentheses

| Approach | MRSE Fig 5A | MRSE Fig 5B | MRSE Fig 5C | MRSE Fig 5D |
|---|---|---|---|---|
| eSMC2 | 9.18 (0.004) | 9.18 (0.004) | 9.44 (0.034) | 9.44 (0.034) |
| SMCm (2%) | 9.34 (0.013) | 9.22 (0.037) | 9.79 (0.057) | 9.59 (0.034) |
| SMCm (10%) | 9.31 (0.07) | 9.12 (0.035) | () | () |
| SMCm (20%) | 9.26(0.011) | 9.07 (0.023) | () | () |
| Approach | MRSE Fig 5A (gen<400) | MRSE Fig 5B (gen<400) | MRSE Fig 5C (gen<400) | MRSE Fig 5D (gen<400) |
| eSMC2 | 7.12 (0.098) | 7.12 (0.098) | 7.01 (0.12) | 7.01 (0.12) |
| SMCm (2%) | 6.46 (0.040) | 4.27 (0.13) | 6.12 (0.17) | 5.00 (0.12) |
| SMCm (10%) | 6.41 (0.045) | 4.43 (0.14) | () | () |
| SMCm (20%) | 6.51 (0.043) | 4.48 (0.10) | () | () |

**Supplementary File 1g.** Average mean root square error of coalescent time along the genome inference. Average mean root square error of inferred coalescent time (in generation unit) along the genome over ten repetitions by the three approaches (eSMC2, SMCm with unknown epimutation rates and SMCm with known epimutation rates) under the same scenario from Figure 5. Inference was performed on two haploid sequences of 10 Mb with $\mu = 7 \times 10^{-9}$, $r = 3.5 \times 10^{-8}$ per generation per bp. Methylation and demethylation rates were respectively fixed to $3.5 \times 10^{-4}$ and $1.5 \times 10^{-3}$ per generation per bp. The selfing rate was fixed to 90%. The coefficient of variation is indicated in parentheses

| Approach | Mean Root Square Error |
|---|---|
| eSMC2 | 46,354 (0.20) |
| SMCm (known epimutation rates) | 46,317 (0.20) |
| SMCm (unknown epimutation rates) | 49,433 (0.22) |

**Supplementary File 1h.** Average mean root square error of demographic inference in Figure 5 - supplement figures 1, 2. Average mean root square error (in log10) of demographic inference in Figure 5 - sup. 1 and 2 by the three approaches (eSMC2, SMCm with unknown epimutations rates and SMCm with known epimutation rates). Note that the second row indicates the MRSE in recent times (younger than 400 generations ago). The coefficient of variation is indicated in parentheses

| Approach | MRSE Fig 5 Sup1A | MRSE Fig 5 Sup1B | MRSE Fig 5 Sup2A | MRSE Fig 6 Sup2B |
|---|---|---|---|---|
| eSMC2 | 9.21 (0.006) | 9.21 (0.006) | 9.22 (0.010) | 9.22 (0.010) |
| SMCm (2%) | 9.26 (0.007) | 9.30 (0.014) | 9.27 (0.017) | 9.59 (0.034) |
| Approach | MRSE Fig 5 Sup1A (gen<400) | MRSE Fig 5 Sup1B (gen<400) | MRSE Fig 5 Sup2A (gen<400) | MRSE Fig 5 Sup2B (gen<400) |
| eSMC2 | 7.21 (0.10) | 7.21 (0.10) | 7.34 (0.065) | 7.34 (0.065) |
| SMCm (2%) | 6.21 (0.046) | 4.87 (0.008) | 6.41 (0.048) | 4,72 (0.064) |

**Supplementary File 1i.** Average mean root square error of demographic inference in Figure 5 - supplement figure 3. Average mean root square error (in log10) of demographic inference in Figure 5 - supplement figure 3 by the three approaches (eSMC2, SMCm with unknown epimutations rates, SMCm with known epimutation rates). Note that the second row indicates the MRSE in recent times (younger than 400 generations ago). The coefficient of variation is indicated in parentheses

| Approach | MRSE Fig 5 Sup3A | MRSE Fig 5 Sup3B |
|---|---|---|
| eSMC2 | 9.19 (0.002) | 9.19 (0.002) |
| SMCm (2%) | 9.19 (0.005) | 9.75 (0.16) |
| Approach | MRSE Fig 5 Sup3A (gen<400) | MRSE Fig 5 Sup3B (gen<400) |
| eSMC2 | 7.27 (0.08) | 7.27 (0.08) |
| SMCm (2%) | 5.88 (0.03) | 9.61 (0.18) |

**Supplementary File 1j.** Average mean root square error of demographic inference in Figure 5 - supplement figure 4. Average mean root square error (in log10) of demographic inference in Figure 5 - supplement figure 4 by the three approaches (eSMC2, SMCm with unknown epimutations rates, SMCm with known epimutation rates). Note that the second row indicates the MRSE in recent times (younger than 400 generations ago). The coefficient of variation is indicated in parentheses.

| Approach | MRSE Fig 5 Sup4A | MRSE Fig 5 Sup4B |
|---|---|---|
| eSMC2 | 9.19 (0.002) | 9.19 (0.002) |
| SMCm (2%) | 9.15 (0.007) | 9.47 (0.007) |
| Approach | MRSE Fig 5 Sup4A (gen<400) | MRSE Fig 5 Sup4B (gen<400) |
| eSMC2 | 7.27 (0.08) | 7.27 (0.08) |
| SMCm (2%) | 5.15 (0.04) | 4.60 (0.02) |

**Supplementary File 1k.** Average estimated rate of the site methylation and demethylation rates in *A. thaliana*. Average estimated values of the site methylation and demethylation rates by SMCm using genomes and methylomes from 10 German accessions of *A. thaliana*. We use eight scaffolds each of 10 sequences with recombination and mutation rate respectively set to $r = 3.6 \times 10^{-8}$ and $\mu_1 = 6.95 \times 10^{-9}$ per generation per bp with selfing set to $90\%$. The polymorphic SMPs CG sites estimations corresponds to the green line in Figure 6. All CG sites estimations and CG site separated by 3,000bp corresponds to the data of the green line in Figure 6 - supplement figure 1.

| Methylation sites chosen | Estimated site methylation rate | Estimated site demethylation rate |
|---|---|---|
| polymorphic SMPs CG sites | $4.4 \times 10^{-5}$ | $5.9 \times 10^{-5}$ |
| CG sites separated by 3,000bp | $2.5 \times 10^{-7}$ | $2.1 \times 10^{-6}$ |
| all annotated CG sites | $4.4 \times 10^{-7}$ | $2.1 \times 10^{-6}$ |

**Supplementary File 1l.** Average estimated rate of the site and region methylation and demethylation rates in *A. thaliana*. Average estimated values of the site and region methylation and demethylation rates by SMCm using genomes and methylomes from 10 German accessions of *A. thaliana*. These estimations are produced during the inference of the red line in Figure 6 - supplement figure 1. We use eight scaffolds each of 10 sequences with recombination and mutation rate respectively set to $r = 3.6 \times 10^{-8}$ and $\mu_1 = 6.95 \times 10^{-9}$ per generation per bp with selfing set to $90\%$.

| Methylation | Estimated methylation rate | Estimated demethylation rate |
|---|---|---|
| sites | $9 \times 10^{-8}$ | $4.9 \times 10^{-7}$ |
| regions | $2.0 \times 10^{-7}$ | $7.3 \times 10^{-7}$ |
